# Fishing fleets as ecosystem sentinels

**DOI:** 10.1073/pnas.2516308122

**Published:** 2025-12-22

**Authors:** Heather Welch, Brett M. Holycross, Allison A. Cluett, Michael G. Jacox, Caren E. Braby, Matthew W. Callahan, Joshua A. Cullen, Nima Farchadi, Rachel Seary, Jordan T. Watson, Steven J. Bograd, Elliott L. Hazen

**Affiliations:** ^a^Institute of Marine Science, University of California, Santa Cruz, CA 94064; ^b^Pacific States Marine Fisheries Commission, Portland, OR 97219; ^c^Institute for Ecological Monitoring and Management, San Diego State University, San Diego, CA 92182; ^d^Biology Department, Woods Hole Oceanographic Institution, Woods Hole, MA 02543; ^e^Durrell Institute of Conservation and Ecology, School of Natural Sciences, University of Kent, Canterbury CT2 7NR, United Kingdom; ^f^Pacific Islands Ocean Observing System, Honolulu, HI 96822; ^g^Pacific Region, Canadian Integrated Ocean Observing System, Victoria BC V8N 1V8, Canada

**Keywords:** ecosystem sentinel, fisheries, climate change, marine heatwave, highly migratory species

## Abstract

Marine apex predators are promising sentinels for detecting the ecological impacts of climate variability and change. Fishermen are increasingly recognized as marine apex predators, and there are extensive satellite-based geolocation data on fishing vessel activities. Despite this potential, the utility of fishermen as ecosystem sentinels remains unexamined. Using one million vessel positions from 600 U.S. vessels, we assess the effectiveness of fishermen as sentinels for the ecological impacts of Northeast Pacific marine heatwaves on tuna distribution and availability. Fishermen were skillful predictors of extreme northward shifts for albacore and bluefin tunas, and extreme inshore shifts for albacore. Fishermen signaled low albacore availability over a year in advance of a formal fisheries disaster declaration request. Notably, fishermen also indicated true negatives during marine heatwaves: periods of anomalous warming but stable tuna distribution and availability. This information could aid management of transboundary shifts during marine heatwaves of albacore from U.S. to Canadian waters and bluefin from Mexican to U.S. waters. Advanced warning of fisheries disasters could expedite the delivery of relief funds for struggling communities. The number of Earth-orbiting satellites is exponentially rising, generating a wealth of geospatial information on fishing vessels. This rich and growing resource can signal otherwise unobserved ecological impacts, aiding rapid management responses to climate extremes.

## Significance

Vessel Monitoring Systems (VMS) data offer near real-time information on where fishing vessels are. It is used by authorities around the world to enforce compliance with fishing regulations. Here, we explore an alternative application for these data: to detect climate impacts on ecologically and economically valuable target species. We find that VMS provides an accurate, real-time indicator of the impacts of marine heatwaves on the location and availability of north Pacific albacore and bluefin tuna. These findings are intuitively credible, since fishing profitability depends on accurately locating and accessing target species. Despite this potential, VMS data are not currently used for operational ecological monitoring. As ocean conditions change, timely ecological information is essential for managing and conserving living marine resources.

Climate variability and change are reshaping marine ecosystems in ways that are challenging to observe directly. While there are many data streams for observing environmental change (e.g., satellites, ocean models, gliders), there are fewer options for observing ecological change. Apex predators are increasingly proposed as tools for monitoring ecological changes that are otherwise difficult to observe, i.e. ecosystem sentinels (1–3). For example, elephant seals are promising sentinels of productivity in the ocean twilight zone (4), and polar bears are considered sentinels of seal body condition (5). Effective sentinels have strong and timely responses to environmental change and are conspicuous (1, 2), i.e., easy to collect data on and visually observe.

Fishermen are increasingly recognized as marine apex predators (6–8), and have many of the qualities of effective ecosystem sentinels. Due to the tight coupling between ecosystem condition and economic productivity (9), fishermen are highly tuned to environmental variability and change (6, 10–14). Fishermen must constantly adapt to climate-driven changes in target species abundance and distribution to maintain profitable fishing opportunities (15). This ecological sensitivity is acquired via direct observation of the physical environment and local ecological knowledge (6, 16). Fishermen can have wide-ranging movements (17, 18) allowing them to effectively sample large portions of the seascape. Importantly, fishermen are conspicuous and their activities are actively monitored via several near real-time, high-resolution data streams including vessel tracking systems, satellite mapping, and shoreside landing receipts (18–20).

Long-term warming (i.e. climate change) and short-term warming events associated with El Niño and marine heatwaves can drive shifts in the distributions of fisheries target species following optimal temperature conditions (21–25). These shifts create geographic redistributions of fishing opportunity and economic potential, which can lead to social conflicts, lack of infrastructure preparedness, and overfishing (9, 26–30). A classic example is the Mackerel War: warming waters shifted mackerel into Icelandic waters in 2007, causing conflicts with Europe over Icelandic fishing quotas (28). A 2012 heatwave in the Gulf of Maine drove lobsters into shallow waters, leading to record catch that outpaced processing capacity and consumer demand (27). Lobster prices fell to 70% below normal and the market crashed. Long- and short-term warming have also led to major fishery collapses, including Bering sea snow crab (29), Gulf of Maine cod (9), and lobster in southern New England (30). A failure to detect climate-driven ecological change—and take action when change is detected—can exacerbate warming impacts. For example, failure to recognize the impact of long-term warming on Gulf of Maine cod resulted in fishing quotas that overestimated population levels and ultimately led to overfishing (9). Insights from ecological sentinels could accelerate management action to mitigate the socioeconomic and ecological consequences of ocean warming.

**Fig. 1.**
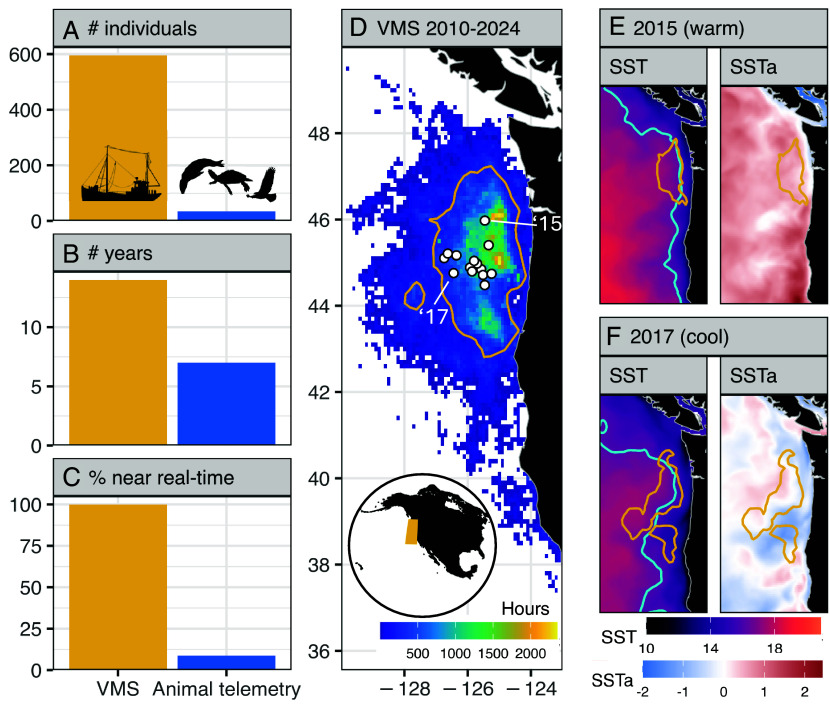
Vessel Monitoring Systems (VMSs) data contain a wealth of information. (*A*–*C*) The dimensions of VMS dataset from the U.S. West Coast Pacific albacore fishery (yellow) compared to the average dimensions of publicly visible animal telemetry datasets in Movebank (blue). (*A*) Number of individual vessels/animals; (*B*) number of years per dataset; (*C*) the percentage of VMS data that update in near real-time (i.e. within the past week) versus the percentage of animal telemetry datasets that update in near real-time. (*D*–*F*) Albacore preferred temperature conditions and fishing effort shift northward and inshore during warm water conditions. (*D*) Total fishing effort (hours) indexed from VMS by the U.S. West Coast Pacific albacore fishery from the 2010–2024 fishing seasons (July–October) mapped at 0.1°. Annual centroids are overlaid as white points with two years labeled (2015, 2017); the inset shows the location of VMS data in yellow. (*E* and *F*) Sea surface temperature (SST) and SST anomalies (SSTa) for the 2015 and 2017 fishing seasons; cyan SST contours at 17 °C mark the lower limit of albacore preferred temperature conditions. Yellow contours in D-F capture the 75th percentile of fishing effort across each time period.

We assessed the potential of commercial fishermen to serve as sentinels for climate-driven ecological changes in target species using Vessel Monitoring System (VMS) data (Fig. 1). VMS is a government-mandated vessel tracking system that broadcasts vessel location and identity information in near real-time, enabling high-resolution spatiotemporal tracking of fishing vessel activity (16, 31–33). In ecological research, the closest analog to VMS data is animal telemetry, which is frequently used to evaluate and describe ecosystem sentinels (4, 31, 34, 35). However, VMS datasets differ from animal telemetry in several critical ways: They typically encompass orders of magnitude more individuals, span longer and more continuous time series, and offer near real-time data availability (Fig. 1 *A*–*C*). Additionally, VMS can be linked to near real-time shoreside landing receipts to provide information on catch composition during fishing trips (36). These features position VMS as a powerful yet underutilized tool for detecting climate-driven ecological change.

We analyzed VMS data linked to shoreside landing receipts (*hereafter*: VMS) from 2010–2024 for the U.S. West Coast Pacific albacore fishery (Fig. 1*D*) to evaluate the suitability of fishermen as sentinels for two ecological changes during anomalously warm conditions: 1) northward and inshore shifts of albacore (*Thunnus alalunga*) and bluefin (*Thunnus orientalis*) tuna, and 2) low albacore availability, with a particular interest in the 2023 season that led to a formal request for a federal fishery disaster declaration (37). Albacore and bluefin are migratory temperate tunas that are seasonally targeted by U.S. West Coast fishermen during the summer and fall (38–40). Both species have been described shifting northward and inshore during warm water conditions (22–24, 26, 39), and exploration of interannual variation in fishing effort reveals the same phenomenon (Fig. 1 *D*–*F*). Although the fishery does not target bluefin, we hypothesized that shifting vessel distributions following albacore could convey information about relative distributional shifts in bluefin. The fishery requested a federal disaster declaration for the 2023 season, citing that anomalously warm ocean temperatures (*SI Appendix*, Fig. S1) caused albacore to become more dispersed than usual, ultimately making them cost-prohibitive to target. The request was made in December 2024, over a year after the 2023 season ended. Real-time inferences from VMS on shifting tuna distribution and availability would offer actionable insights for fisheries management—informing decisions related to port operations, overfishing risk, and potential economic impacts. In this period of rapid environmental variability and change, rapid ecological information is vital for timely and responsive decision-making.

## Results

### Northward and Inshore Shifts of Tunas.

We used SEDI (Symmetric Extremal Dependence Index) to evaluate the correspondence between extreme northward and inshore shifts in fishing effort (as indexed by VMS) and extreme northward and inshore shifts in albacore and bluefin tuna habitat [as indexed by species distribution models (21)]. We used the 70 to 95th percentiles over 0.05 increments (n = 6) to define extreme northward and inshore shifts in VMS, albacore and bluefin tuna, and extreme warm sea surface temperature anomalies (SSTa). For example at the 90th percentile, this configuration measures the relative skill of the upper 10% of northward VMS shifts vs. the upper 10% of warm SSTa at predicting the upper 10% of northward albacore habitat shifts. Increasing the percentile (e.g., from 70 to 75%) captures how skill changes as shifts become more extreme.

**Fig. 2.**
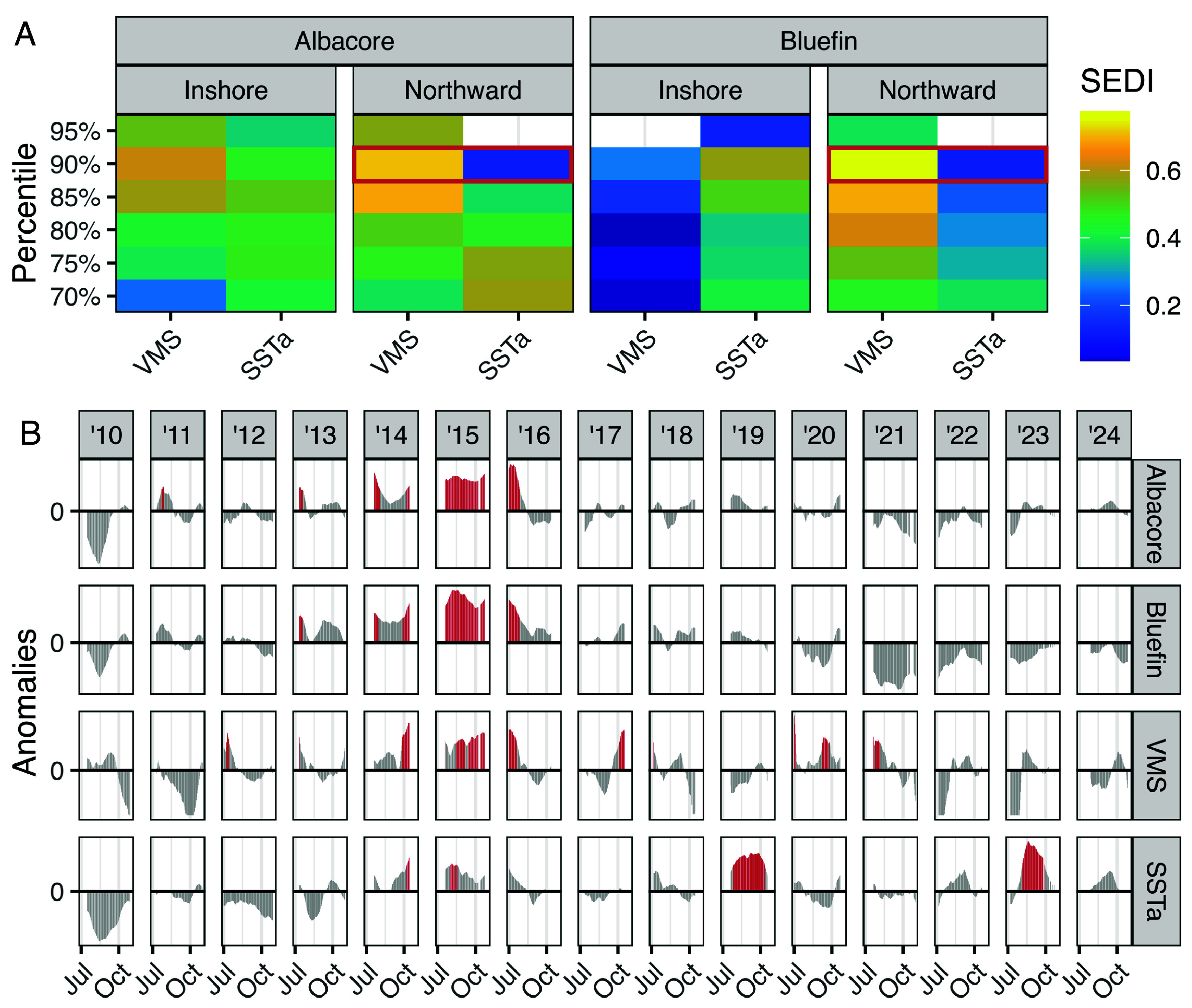
Fishermen are skillful sentinels of northward and inshore shifts of albacore and northward shifts of bluefin. (*A*) SEDI (Symmetric Extremal Dependence Index) is a measure of predictor skill relative to random; SEDI values greater than zero indicate skill better than random. Panels show SEDI values for two daily predictors (fishermen indexed by the VMS and sea surface temperature anomalies; SSTa) versus modeled daily albacore and bluefin inshore and northward habitat shifts. Percentiles (y-axis) are used to constrain data to extreme shifts, e.g., the 90th percentile evaluates the skill of the upper 10% of extreme VMS northward shifts at predicting the upper 10% of extreme albacore northward shifts. Blank tiles indicate worse than random SEDI values; red boxes correspond to time-series in panel *B*. (*B*) Daily latitudinal anomalies indicating north/south movements for modeled albacore and bluefin tunas and VMS, and daily SSTa. The 90th percentile (reds) was used to define extreme northward shifts in albacore, bluefin, and VMS, and extreme positive SSTa.

VMS performed better than random (SEDI > zero, see methods) with the exception of predicting the most extreme shifts (95th percentile, Fig. 2*A*). VMS outperformed SSTa (a simple environmental proxy) at predicting: 1) extreme albacore inshore habitat shifts ≥85th percentile, 2) extreme albacore northward habitat shifts ≥80th percentile, and 3) all extreme bluefin northward habitat shifts. VMS conferred more advantage over SSTa at predicting extreme northward habitat shifts than extreme inshore habitat shifts. For example, VMS was six times better than SSTa at predicting extreme bluefin northward habitat shifts at the 90th percentile (SEDI values of 0.77 and 0.12 for VMS and SSTa respectively, Fig. 2*A*). This was due to the co-occurrence of extreme northward shifts in VMS and tuna habitats during a 2014-2016 large marine heatwave event (41) (Fig. 2*B*). Although SSTa was positive during this period, the majority of extreme positive SSTa events occurred during 2019 and 2023, when VMS and tuna habitats did not exhibit extreme northward shifts.

Using VMS to predict extreme tuna northward shifts would have resulted in true positives during 2014-2016 (extreme shifts were predicted, and occurred), and true negatives in 2019 and 2023 (extreme shifts were not predicted, and did not occur). Using SSTa to predict extreme northward tuna habitat shifts would result in false negatives during 2014-2016 (extreme shifts were not predicted, but did occur), and false positives in 2019 and 2023 (extreme shifts were predicted, but did not occur).

Fishermen consider numerous ecological, economic, and social factors to make decisions about where and when to fish (6, 16). Consequently, latitudinal and inshore/offshore shifts of VMS (Fig. 1 *D*–*F* and 2) may reflect alternative drivers like market fluctuations alongside changing ecological conditions. We used multiple regression models to assess the standardized effect size (β) between latitudinal and inshore/offshore shifts of VMS and two ecological drivers: latitudinal and inshore/offshore shifts of albacore, SSTa; as well as three alternative drivers: area (km^2^) of weather alerts, marine fuel price per gallon (USD), albacore price per pound (*SI Appendix*, Table S1). Latitudinal shifts of albacore habitat had the greatest effect on latitudinal shifts of VMS (β = 0.36, *P* < 0.001) followed by fuel price (β = –0.16, *P* < 0.001). Inshore/offshore shifts of albacore habitat had the greatest effect on inshore/offshore shifts of VMS (β = 0.27, *P* < 0.001) followed by SSTa (β = –0.18, *P* < 0.001). While the distribution of VMS is influenced by both ecological conditions and alternative drivers, its responsiveness to shifts in albacore distribution and ocean temperature suggest it is a useful proxy for detecting ecological change.

### Low albacore availability in 2023.

**Fig. 3.**
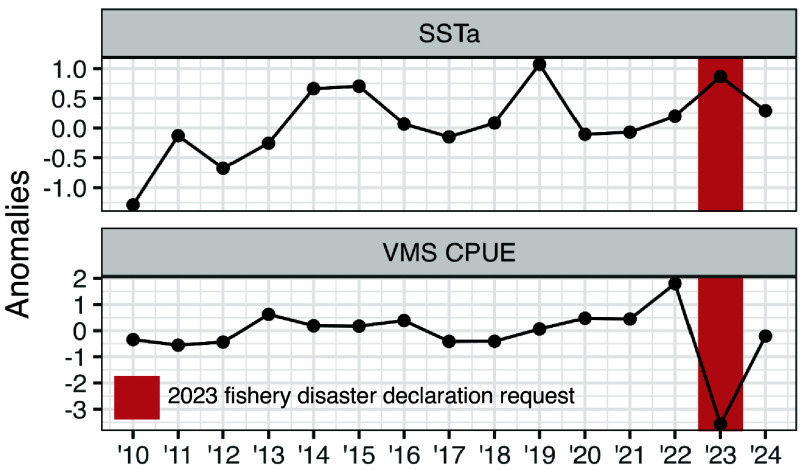
Fishermen are skillful sentinels for a federal fishery disaster declaration request. The U.S. West Coast Pacific albacore fishery requested a federal fishery disaster declaration for the 2023 season, citing low albacore availability due to anomalously warm temperatures. Plots show annual sea surface temperature anomalies (SSTa) in the fishing grounds (*Top*) and annual albacore catch per unit effort (CPUE in units of pounds per minute) indexed by the Vessel Monitoring System (VMS; *Bottom*). Annual data points are averages calculated over the July–October fishing season, and thus VMS CPUE inferences become available in October of each year.

We evaluated the ability of an annual time-series of albacore catch per unit effort (CPUE; in units of pounds per minute indexed by VMS) anomalies to reflect low albacore availability during the 2023 fishing season, and the subsequent fishery disaster declaration request. As for albacore and bluefin distribution shifts, we compared the performance of VMS CPUE to SSTa within the fishing grounds to evaluate whether a simple environmental indicator could convey the same information as a relatively complex indicator. VMS CPUE was quite consistent across years except for the 2023 season during which it decreased by 46% (–3.6 pounds/minute), contrasting with anomalously warm SSTa during the 2014, 2015, 2019, and 2023 seasons (Fig. 3).

## Discussion

We demonstrate that fishermen are promising sentinels for elucidating climate-driven impacts on target species. Daily variability in VMS-derived fishing effort by the U.S. West Coast Pacific albacore fishery revealed patterns of northward and inshore shifts of albacore tuna, and northward shifts of bluefin tuna (Fig. 2). Interannual variability in VMS-derived albacore CPUE reflected low albacore availability during the 2023 fishing season, which prompted a fisheries disaster declaration request (Fig. 3). Notably, these ecological changes were up to six times more strongly predicted by VMS than by SSTa. The northeast Pacific has experienced frequent warming events since the 2014–2016 large marine heatwave (21, 41), however only some events triggered pronounced ecological responses. Tuna distribution and availability are shaped by a suite of environmental variables and a complex set of ecological processes such as prey availability and population structure (21, 25, 38), which SSTa alone cannot fully capture. While SSTa is widely accessible and easy to compute, our results suggest it is an overly simplistic proxy for ecosystem change. In contrast, VMS data integrate the behavioral responses of fishermen to a broad suite of environmental and ecological signals, providing a sensitive and accurate indicator of tuna distribution and availability.

There is an inherent paradox in evaluating the efficacy of ecosystem sentinels: the very conditions that make ecosystem sentinels necessary—limited direct observations of ecological change—also make it difficult to ground-truth their effectiveness. Here, we used correlative species distribution models (SDMs) for albacore and bluefin tuna to ground-truth the effectiveness of fishermen as ecosystem sentinels. SDM predictions have advantages over other species occurrence datasets like animal telemetry in that information on species distributions is spatially and temporally contiguous, and less biased toward the locations or individuals selected for tagging. But similar to SSTa, correlative SDMs do not explicitly capture the ecological processes that affect species distributions (21). In addition, correlative SDMs assume static species–environment relationships, which can result in skill decay under novel environmental conditions (42). While the models themselves can be operationalized to provide real-time inferences on tuna distribution (43), VMS-based indicators may offer an advantage by capturing integrated ecological responses and by responding to current environmental conditions. SDMs and VMS-based indicators could be integrated to couple forecasts of future conditions with observations of present conditions, analogous to approaches in weather forecasting.

Fisheries-dependent datasets such as VMS are subject to preferential sampling, wherein fishermen concentrate effort in areas where they expect to encounter target species. Preferential sampling can lead to biased inferences on target species distributions. Future work could consider methods that jointly model ecological and sampling processes to implicitly address bias (44, 45). Using this method, near real-time inferences on species distributions could be produced operationally by predicting the joint process model over new environmental covariates for each day. Additionally, the joint process model itself could be updated each day as new VMS and landings data become available.

### Management Implications.

The North Pacific albacore population is considered healthy and the U.S. commercial fishery is not subject to quotas (46). A portion of U.S. vessels fish and land catch in Canada as allowed under provisions of the 1981 U.S.-Canada Albacore Treaty (47). Real-time information on the northward shifts of albacore could provide insights into northward shifts in port usage and processing infrastructure needs, and help anticipate increased U.S. fishing effort in Canadian waters. Information on transboundary albacore shifts is especially important because Canada and the United States do not always reach annual access agreements, and limited access during northward shift events could substantially impact U.S. fishing opportunities. Real-time information on inshore shifts of albacore could signal increased catch by small commercial and recreational vessels, which operate closer to shore than larger commercial vessels. Elevated catch can flood the market and drive down price, as occurred during the 2012 heatwave-driven crash of the lobster market in the Gulf of Maine (27). Temporary catch limits or effort controls could be implemented to avoid market collapse.

The fishery requested a disaster declaration for the 2023 season in December 2024, more than a year after the season concluded (37). Yet, interannual variability in albacore CPUE suggested in October 2023 that a request was likely. On average, U.S. West Coast fisheries submit disaster declaration requests 380 d after the affected season (*SI Appendix*, Fig. S2), likely due to the time required to compile supporting information. Early indicators of poor fishing conditions could enable earlier detection of potential disasters and delivery of relief funds to struggling communities. As apex predators, albacore may also aggregate information on the status of the forage base. Albacore prey on economically important species like sardine, anchovy, rockfish, and squid (25, 48). Indicators of albacore distribution and availability could provide valuable insights into the health and trajectory of these other fisheries.

Our findings suggest that shifts in vessel distributions following albacore also reflect relative changes in bluefin distribution. Both tunas exhibit similar responses to anomalous warming (22–24), and warming events often cover large spatial areas, affecting the distributions of both tunas concurrently (21). Pacific bluefin tuna have made a notable recovery due to coordinated basin-scale conservation efforts. From precipitously low abundance in the 1990s and petition for endangered status in 2016, today bluefin are considered healthy (49). The U.S. bluefin quota for 2025–2026 reflects this rebound, with an 80% increase over the previous quota (50). As fishing interest grows, this emergent resource will need careful monitoring to ensure quotas are not exceeded, which has occurred previously (26). Bluefin move north–south seasonally along the North American west coast, inhabiting Mexican waters in the spring and U.S. waters in the fall (51). Real-time information on northward shifts would provide early insights into increased bluefin availability to US fishermen. These insights could be used to temporarily pause bluefin fishing until landings are fully counted to avoid quota overages and overfishing.

### Advancing the Field.

**Fig. 4.**
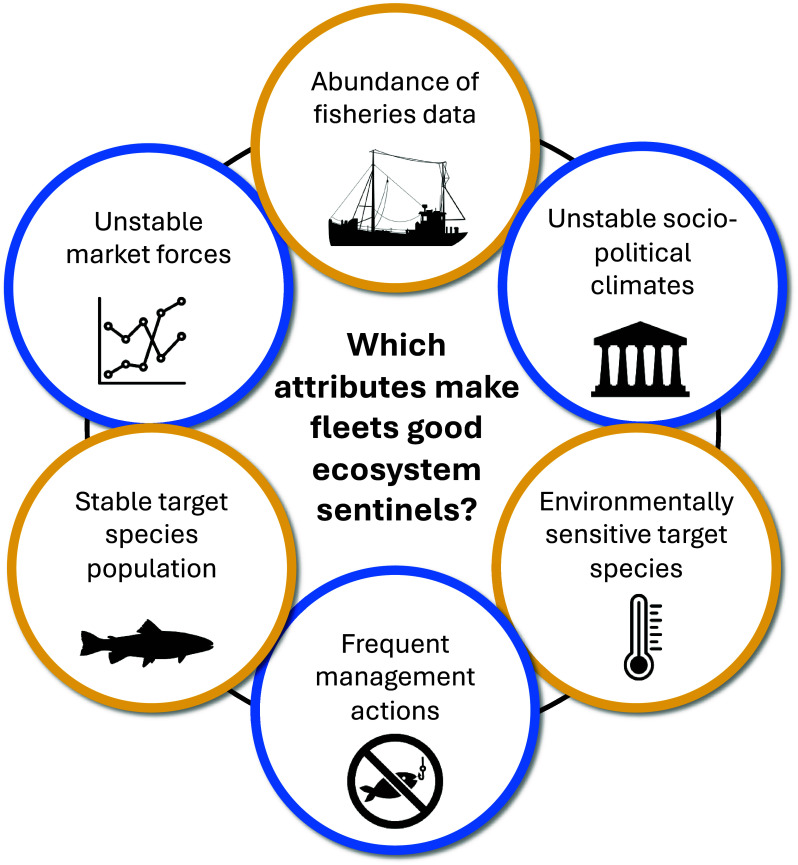
Fleets that make good ecosystem sentinels have high signal-to-noise ratios. Yellow circles represent signals and blue circles represent noise, i.e. attributes to target and avoid respectively when evaluating candidate sentinel fleets. An abundance of fisheries data (e.g., many vessels broadcasting their location), environmentally sensitive target species, and stable target species populations (e.g., without natural boom-bust cycles) create strong signals. Unstable sociopolitical climates (e.g., wars, human health crises) and market forces (e.g., consumer demand, seafood price per pound) create noise, as do fisheries management actions like fishing closures and gear reductions.

Effective ecosystem sentinels have a high signal-to-noise ratio, meaning they respond predictably and measurably to ecosystem changes of interest (signal), with minimal interference from confounding factors (noise). For example, signals of fluctuating twilight zone productivity derived from elephant seals (4) may be confounded by human disturbance at the rookery, disease, or pollution-related declines in body condition. Here, we assessed whether signals of shifting albacore distributions from VMS were confounded by adverse weather, marine fuel prices, and the market value of landings (*SI Appendix*, Table S1). Below we outline attributes to target and avoid when evaluating candidate sentinel fleets to maximize the signal-to-noise ratio, while acknowledging that noise cannot be eliminated entirely (Fig. 4).

*Signal*. Individual vessels have different fishing strategies, capabilities, and decision-making processes (16). A large dataset helps average out this vessel-specific noise, allowing the ecological signal to emerge more clearly. For example, adverse weather may force nearby vessels to fish close to shore, but data from unaffected vessels elsewhere can smooth out this localized pattern. Fleets that target environmentally sensitive species are likely to have stronger ecological signals than fleets that target less responsive species. Tuna are highly mobile and shift their distributions substantially in response to anomalous warming (22, 24). In contrast, anomalous warming has relatively little influence on the distribution of demersal fishes (52) and their fisheries would be less likely to reflect a warming signal. Fleets that operate as central-place foragers have less capacity to shift their distributions compared to nomadic fleets, and thus changes in CPUE may provide a more reliable signal of shifting target species distributions. Species’ populations have different trajectories: Albacore are stable (46), bluefin are increasing due to management intervention (49), and sardines have a natural boom-bust cycle (53). The ecological signal can be confounded by population trajectory, e.g., CPUE of bluefin and sardine is likely to reflect management intervention and the natural boom-bust cycle, respectively. Fleets that target species with stable populations have more potential to indicate climate-driven ecological processes.

*Noise*. Sources of noise can introduce spurious data points that weaken the ecological signal or, if strong enough, generate misleading signals of their own. Fleets that operate in areas with unstable sociopolitical climates may have noise from sources like wars, political unrest, or human health crises. For example, signals from the COVID-19 pandemic are apparent in vessel behaviors (54), as are signals from piracy and maritime boundary conflicts (55). Frequent fisheries management actions like closures, quota changes, and gear or depth restrictions also affect vessel behavior and generate noise (56). Fleets that operate in international waters are generally subject to fewer management actions relative to domestic fleets and thus have high potential to serve as sentinels. The albacore fishery is open-access, meaning that there are no restrictions on participation, catch, or fishing location and timing. However, the United States and Canada do not always reach annual access agreements, which could weaken the signal of northward shifts. Unstable market forces affect how fishermen navigate cost–benefit trade-offs in deciding where, when, and what to fish for. For example, high marine fuel prices may drive fleets to fish close to shore, and low market value or consumer demand may drive fleets to change target species, or not fish at all. Another attribute to consider is the number of species fleets target. Single-species fleets may provide cleaner signals, as multispecies fleets can have noise from differing market forces or environmental sensitivities across species, for example. However, multispecies fleets may capture signals that reflect a more integrated view of ecosystem condition by aggregating information across species. The tradeoffs between single- and multispecies fleets mirror those of specialist versus generalist animal sentinels: Specialists yield cleaner signals due to their narrow ecological niches, whereas generalists can capture broad changes in the prey base (1).

Consideration of the signal-to-noise ratio can help practitioners identify fleets with high potential to reflect ecological signals, and provides priors for interpreting spurious signals. Nevertheless, human and animal sentinels will always have false positives and negatives. For example, California sea lions have been proposed as sentinels of anomalous warming impacts: Colonies failed during warm water events in 1997-98 and 2014-15, but also failed in 2012 when temperatures were normal (57). In our increasingly automated and data-driven world, human oversight is critical to prevent noise from causing harm. This human-in-the-loop approach involves system and species experts reviewing inferences from sentinels before it is translated into management action. The volume, quality, and immediacy of fisheries datasets is rapidly increasing (58). A precautionary approach allows this rich informationscape to be harnessed for ecological observation and nimble decision-making.

## Materials and methods

### VMS Linked to Albacore Landings.

VMS data contain information on vessel movements in space and time, but do not include information on which species vessels catch. In contrast, landings receipts contain information on which species vessels catch, but do not generally include information on vessel movements. VMS data are captured by vessel-mounted transponders that ping roughly once every hour, although there is some variability. Thus, VMS data provide vessel movement information at high spatiotemporal resolutions for each fishing trip. Landing receipts are port-level records of catch during each vessel’s fishing trip. Linking these two datasets has become a valuable method for investigating where and when vessels catch species of interest (36).

Shore-side landings receipts were accessed from the Pacific Fisheries Information Network (PacFIN; https://pacfin.psmfc.org/about/about-pacfin/) for all days from 2010–2024 for Washington, Oregon, and California, USA. New landings receipts are received, postprocessed, and served by PacFIN daily. Landings receipts included fields for timestamp, vessel number (a unique identifier for each vessel), ticket number (a unique identifier for each landing), species, landed weight of each species in pounds, and the inflation adjusted price per pound for each species. Landing receipts were filtered to those with at least 90% of the total landed weight belonging to albacore (36).

VMS data were linked to albacore landings receipts and filtered to capture albacore fishing activity. VMS was accessed from PacFIN for all days from 2010-2024 for all U.S. vessels in the northeast Pacific. New VMS data are received daily, but are batch postprocessed and served by PacFIN weekly. Raw VMS data include fields for timestamp, vessel number, latitude, and longitude. The following auxiliary fields are added within PacFIN: 1) “event type” (e.g., in port, at-sea, departing port), 2) “time forward” in minutes until the next VMS ping, “speed” in nautical miles (nm) per hour, 3) and “GIS code” (a flag for potential VMS signal breaks or multiple vessels using the same vessel number at the same time). VMS data were filtered by vessel number to vessels with landing receipts with at least 90% of the landed weight belonging to albacore.

The filtered VMS and landings receipt datasets were joined using the timestamp and vessel number fields following methods developed by Feist et al. (36). For a given landing receipt, VMS pings preceding the receipt in time were assigned to the receipt until the shorter of 1) the timestamp of the preceding landing receipt or 2) the 75th percentile of albacore fishing trip lengths (36) (16 d). The 75th percentile controls for cases in which a vessel has two sequential landing receipts that are far apart in time, e.g., several months. The vessel may have at sea activity during this time that is not related to the landing receipt, for example transiting between two ports.

The joined VMS-landings dataset was filtered to remove pings flagged in the “GIS code” field and to at-sea pings in the “event type” field. Pings with “time forward” greater than 121 min (i.e., one missed ping) were removed to further control for signal breaks. Next, a speed filter of 3.74 nm per hour (59) was applied to isolate albacore fishing activity, such that pings with speeds less than 3.74 nm were considered fishing, and pings with faster speeds were considered nonfishing and removed. Then, the landed pounds of albacore in each landing receipt was distributed proportionally to the “time forward” of each VMS ping associated with the landing receipt. For example, if a landing receipt with 1,000 lbs of albacore was associated with two VMS pings with “time forwards” of 30 and 70 min, the pings were assigned 300 and 700 lbs of albacore. The final VMS linked to albacore landings dataset contained 943,000 fishing pings from 596 vessels.

### Indicators.

The final joined VMS-landings dataset was summarized using daily centroids weighted by landed pounds of albacore. For each day, distance to shore was calculated as the mean distance of VMS pings to the coastline weighted by landed pounds of albacore. Daily catch per unit effort (CPUE) in pounds per minute was calculated by dividing the daily sum of landed albacore by the daily sum of “time forward.” Daily anomalies of Y (latitudinal) centroid, distance to shore, and CPUE were calculated relative to the monthly climatology, which is conditions in each month averaged across 2010–2024. The monthly climatology was used instead of a daily climatology to reduce noise inherent in short time-series. For example, in a time-series of 2010–2024 each day has a maximum of 15 values with which to construct a climatology. Daily anomalies were smoothed using a 30-day rolling mean right aligned on each day in the time-series. Last, the time-series were restricted to July–October to match the peak albacore fishing season (*SI Appendix*, Fig. S3). Together, these three time-series capture north/south movement (Y centroid), inshore/offshore movement (distance to shore), and albacore availability (CPUE).

GLORYS gridded daily sea surface temperature (SST, 1/12°) data from 2010–2024 were downloaded from Copernicus Marine Environment Monitoring Service. Gridded SST was masked to the 75th percentile kernel of VMS pings weighted by “time forward” to isolate environmental conditions where the majority of fishing activity occurs [contour in Fig. 1*D*; *sensu* (7, 21)]. To identify the 75th percentile kernel, a two-dimensional bandwidth matrix was estimated using the Hpi plug-in method for anisotropic measures of kernel density estimation. Density estimates were evaluated over the SST grid, normalized, and then ranked to calculate cumulative probability of use per grid cell. Daily SSTa were calculated and smoothed following the same methods used to calculate the VMS anomalies. SSTa was included to investigate whether VMS (a more complicated indicator) conferred any advantage over a simpler environmental indicator.

Boosted Regression Tree (BRT) model predictions of albacore and bluefin tuna distributions (i.e. habitat suitability) (21, 60) were extended to 2023-24 to match the time-series of the VMS-landings dataset. The BRT model predictions are based on 9 dynamic covariates (SST, day of year, SST SD, eddy kinetic energy, sea level anomaly, mixed layer depth, chlorophyll-a, oxygen, and primary productivity) and two static covariates (bathymetry, rugosity). Model predictions were masked to the US Exclusive Economic Zones (61) (EEZ). The predictions cover large portions of the Northeast Pacific (*SI Appendix*, Fig. S4), however most albacore fishing activity occurs off of Oregon and Washington (Fig. 1). This mask focused the analysis on portions of tuna distributions actively fished by U.S. West Coast vessels. The EEZ mask was used instead of the 75th percentile kernel (as with SSTa) to preserve enough area to capture distribution shifts. Core habitat was defined using the 75th percentile of predicted values as a threshold (62–64), such that grid cells with predicted values greater than the threshold were considered core, and grid cells with predicted values less than the threshold were removed. For each species, daily centroids weighted by model predicted values were calculated, and distance to shore was calculated as the mean distance of pixels to the coastline weighted by model predicted values. Daily anomalies of Y centroid and distance to shore were calculated and smoothed following the same methods used to calculate the VMS anomalies.

### SEDI.

The SEDI is a skill metric developed within the field of weather forecasting to measure the ability of forecasts to predict rare events (true positives, TP) and avoid false alarms (false positive, FP) (65). SEDI operates on binary classifications, that is whether or not an event was observed to occur, and whether or not that event was forecast. The formula for SEDI is

$$\begin{array}{c} SEDI=\frac{log\;(F)-log\;(H)-log\;(1-F)+log\;(1-H)}{log\;(F)+log\;(H)+log\;(1-F)+log\;(1-H)}, \end{array}$$

where *H* is the hit ratio calculated as TP/(TP + false negatives), and *F* is the false alarm rate calculated as FP/(FP + true negatives). SEDI scores are bounded by negative one and one, where scores above zero are better than random chance and a score of one indicates perfect prediction. SEDI has several useful properties for this present application (66): 1) it does not trend toward a meaningless limit as event rarity increases (e.g., infinity), and 2) it is not affected by rare event frequency, meaning it provides a stable measure of skill even when the event being predicted is extremely uncommon.

We used SEDI to evaluate the skill of VMS and SSTa to predict extreme northward and inshore shifts of albacore and bluefin tuna habitat (Fig. 2). Although SEDI is not affected by rare event frequency, its interpretation requires caution when applied to short data records. Only a few marine heatwaves occurred during our 15-year time-series, introducing some statistical uncertainty into the SEDI results.

### Secondary Analyses.

Fig. 1. Metadata on the dimensions of public datasets on Movebank (https://www.movebank.org/; Fig. 1 *A*–*C*) were accessed via HTTP request: https://github.com/movebank/movebank-api-doc/blob/master/movebank-api.md#get-a-list-of-studies. Public datasets represent 84% of Movebank datasets. The metadata includes attributes for the number of tagged animals and the timestamps of the first and last deployments (Fig. 1 *A* and *B*). The metadata updates approximately once per day when available, allowing for calculation of percent of datasets that update in near real-time (i.e., within the last week; Fig. 1*C*). Pixels with data from less than four vessels were removed due to confidentiality restrictions (Fig. 1*D*). Removed pixels represent 4% of VMS pings. Albacore preferred temperature conditions (17 to 21 °C; Fig. 2 *E* and *F*) were derived from model partial response curves (21). Waters ≥21 °C do not occur within map domain and time period, and therefore the upper thermal contour is not visible. SSTa was calculated relative to the 2010-2024 climatology (Fig. 2 *E* and *F*).

*SI Appendix*, Fig. S2. U.S. West Coast fisheries disaster declaration requests were compiled from https://www.fisheries.noaa.gov/national/funding-financial-services/fishery-resource-disaster-determinations. Metadata includes the date the request was received (YYYY-MM-DD) and the affected season (YYYY). To calculate the number of days between poor fishing seasons and subsequent fisheries disaster declaration requests (*SI Appendix*, Fig. S2), we assumed that each season ended on YYYY-12-31. This approach is conservative with regard to delay length (i.e. we are underestimating the delay), but avoids making imprecise estimates about fishing season end dates across unfamiliar fisheries.

*SI Appendix*, Table S1. Daily marine fuel prices for ports in Oregon and Washington from 2010-2024 were accessed from the Pacific States Marine Fisheries Commission (https://www.psmfc.org/efin/data/fuel.html#Data) and summarized by average daily price per gallon of #2 marine diesel. Fuel price was adjusted for inflation using a 2017 baseline consumer price index from the U.S. Bureau of Economic Analysis (https://fred.stlouisfed.org/series/GDPDEF). Fuel prices are irregularly collected, and linear interpolation was used to gap-fill missing days. Daily weather alerts off of Oregon and Washington from 2010–2024 were accessed from the Iowa Environmental Mesonet (https://mesonet.agron.iastate.edu/) and summarized by average daily area in alert status (km^2^). Daily inflation adjusted price per pound of albacore landings from Oregon and Washington from 2010–2024 was accessed from PacFIN. Linear interpolation was used to gap-fill missing days. Daily anomalies of fuel price, weather alert area, and albacore price per pound were calculated and smoothed following the same methods used to calculate the VMS anomalies.

Multiple regression models were used to examine the relationship between VMS indicators (Y centroid and distance to shore; responses) and each of the five predictors: albacore indicators (Y centroid and distance to shore), SSTa, fuel price, weather alert area, and albacore price per pound (n = 2 models, *SI Appendix*, Table S1).

## Supplementary Material

Appendix 01 (PDF)

## Data Availability

Code and data to recreate the main and supplementary figures and tables data have been deposited in Zenodo (https://doi.org/10.5281/zenodo.17727863) (67). Confidential vessel-level landings and Vessel Monitoring System data may be acquired by direct request to the US National Marine Fisheries Service Office of Law Enforcement via the Pacific States Marine Fisheries Commission (https://www.psmfc.org/contact/), subject to a non-disclosure agreement.
