# Supplementary material for: Fishing fleets as ecosystem sentinels
Source: Proc Natl Acad Sci U S A. 2025 Dec 22;122(52):e2516308122. doi: 10.1073/pnas.2516308122 (PMC12772164; doi:10.1073/pnas.2516308122)
Supplement: Supplementary file 1 — Appendix 01 (PDF) [file pnas.2516308122.sapp.pdf]

PNAS

Supporting Information for
Fishing fleets as ecosystem sentinels

Heather Welch[1*], Brett M. Holycross[2], Allison A. Cluett[1], Michael G. Jacox[1], Caren E. Braby[2], Matthew W. Callahan[2], Joshua A. Cullen[1], Nima Farchadi[3,4], Rachel Seary[1,5], Jordan T. Watson[6,7], Steven J. Bograd[1], Elliott L. Hazen[1]

[1] Institute of Marine Science, University of California Santa Cruz, Santa Cruz, CA, 94064, U.S.A.
[2] Pacific States Marine Fisheries Commission 6720 S. Macadam, Suite 200, Portland, OR, 97219, U.S.A.
[3] Institute for Ecological Monitoring and Management, San Diego State University, San Diego, CA, 92182, U.S.A.
[4] Biology Department, Woods Hole Oceanographic Institution, Woods Hole, MA, 02543, U.S.A.
[5] Durrell Institute of Conservation and Ecology, School of Natural Sciences, University of Kent, Canterbury, U.K., CT2 7NR
[6] Pacific Islands Ocean Observing System, Honolulu, HI, 96822, U.S.A.
[7] Pacific Region, Canadian Integrated Ocean Observing System, Victoria, B.C., V8N 1V8

[*] Corresponding author; Email: hwelch@ucsc.edu

This PDF file includes:

Figures S1 to S4
Tables S1
SI References

Figures

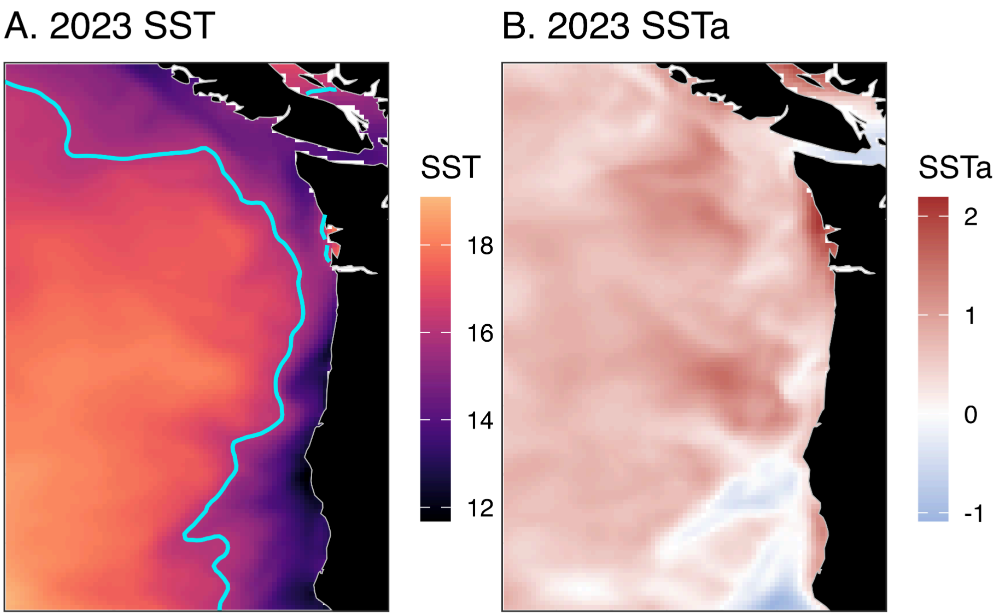

Fig. S1. Sea surface temperature (SST) and SST anomalies (SSTa) for the 2023 fishing season (July-October). Cyan SST contours at 17°C mark the lower limit of albacore preferred temperature conditions. SSTa was calculated relative to the 2010-2024 climatology.

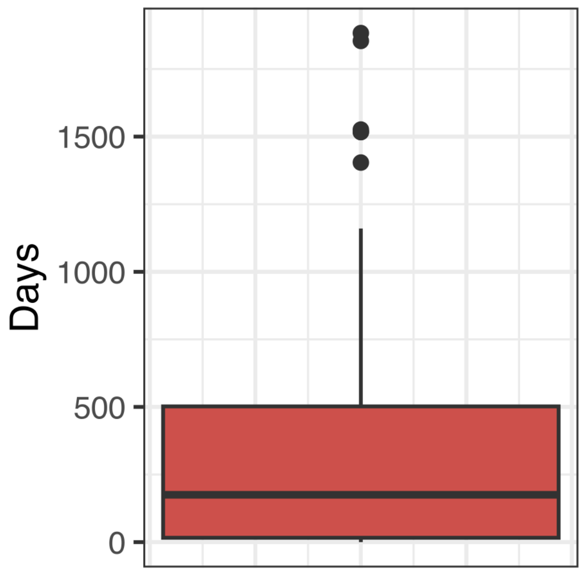

Fig. S2. Number of days between poor fishing seasons and subsequent fisheries disaster declaration requests (mean = 380 days). Data includes 55 disaster requests from U.S. west coast fisheries from 2009-2025 covering 77 seasons (an individual request can cover multiple seasons).

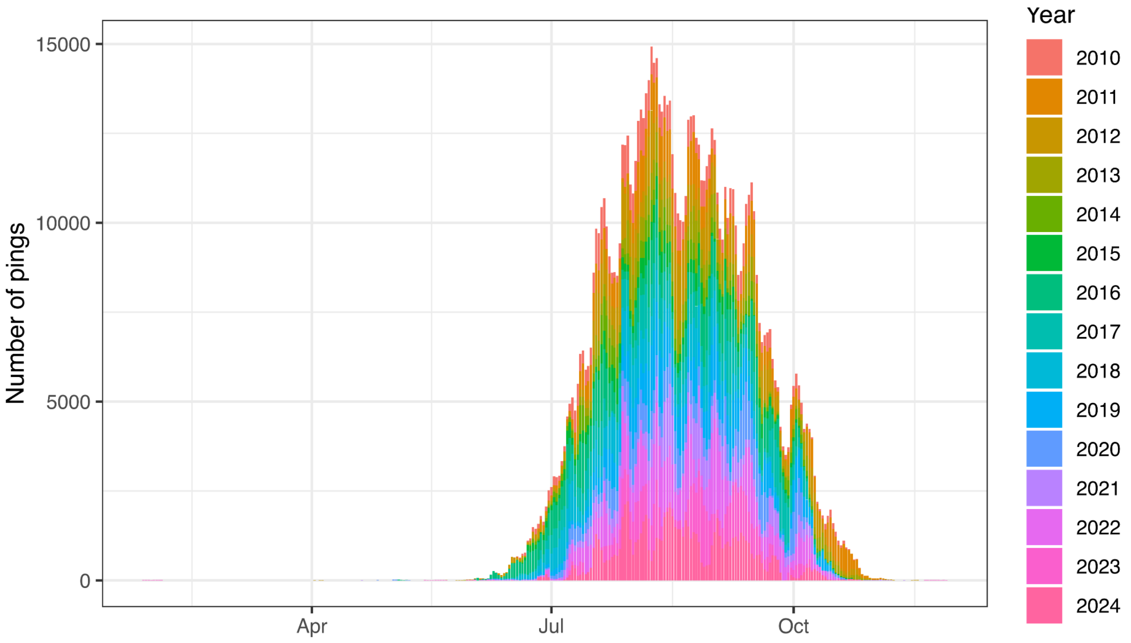

Fig. S3. Number of Vessel Monitoring System (VMS) pings per day 2010-2024 in the joined VMS-landing dataset. VMS indicators (Y centroid, distance to shore, and catch per unit effort) are calculated using a 30-day rolling mean right aligned on each day in the time-series. The time-series were restricted to July-October to match the peak albacore fishing season accounting for the right alignment of the rolling mean.

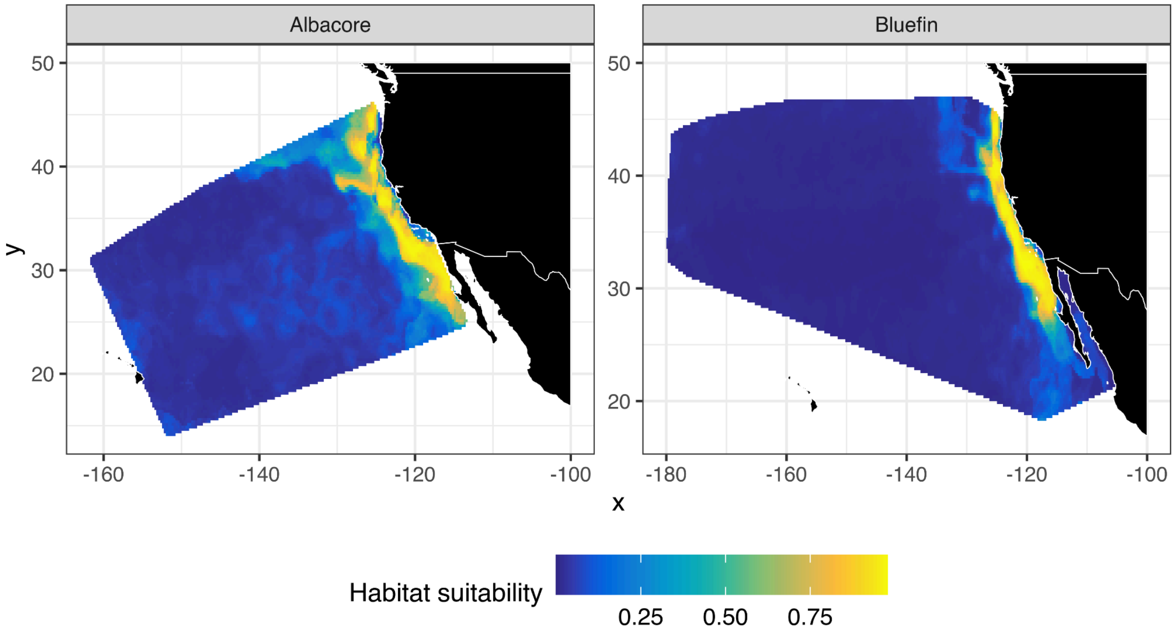

Fig. S4. Boosted regression tree model predictions for albacore and bluefin tuna for an example day (2019-09-01). Prediction domains are constrained by convex hull polygons based on the telemetry datasets used in model fitting (see (1)). This ensures that model predictions are not extrapolated beyond the spatial bounds of the training data.

Tables

Table S1. Multiple regression models used to explore the relationship between Vessel Monitoring System (VMS) indicators (Y centroid and distance to shore; responses) and five predictors: albacore indicators based on boosted regression tree (BRT) models (Y centroid and distance to shore), sea surface temperature anomalies (SSTa), fuel price per gallon, weather alert area, and albacore price per pound (lb).

Response	Predictor	Estimate	Std. Error	t value	P-value
VMS Y centroid	Albacore BRT Y centroid	0.36	0.031	11.6	< 0.001
	Albacore price per lb	-0.09	0.025	-3.38	< 0.001
	Fuel price per gallon	-0.16	0.025	-6.32	< 0.001
	SSTa	-0.07	0.031	-2.38	0.017
	Weather alert area	0.01	0.026	0.27	0.786
VMS distance to shore	Albacore BRT distance to shore	0.27	0.031	8.59	< 0.001
	Albacore price per lb	0.16	0.024	6.56	< 0.001
	Fuel price per gallon	-0.15	0.028	-5.4	< 0.001
	SSTa	-0.18	0.029	-6.33	< 0.001
	Weather alert area	-0.02	0.026	-0.82	0.41

SI References

1. H. Welch, *et al.*, Impacts of marine heatwaves on top predator distributions are variable but predictable. *Nat. Commun.* **14**, 5188 (2023).
